# Biochemical and Hepatic Determinants of Reproductive Failure in Reptiles: A Review of Dystocia Pathophysiology and Management

**DOI:** 10.3390/vetsci13010030

**Published:** 2025-12-27

**Authors:** Margot Morel, Michaela Gumpenberger, Hermann Kempf, Sarah Green, Remco A. Nederlof, Jaco Bakker

**Affiliations:** 1 Broadway Veterinary Group, Unit 1 the Links, Herne CT6 7FE, UK; sarahg@broadwayvetgroup.co.uk; 2 Clinical Department for Companion Animals and Horses, Diagnostic Imaging, University of Veterinary Medicine Vienna, Veterinärplatz 1, 1210 Vienna, Austria; michaela.gumpenberger@vetmeduni.ac.at; 3 Tieraerztliche Praxis fur Exoten, 86167 Augsburg, Germany; hermann.kempf@gmx.de; 4 Independent Researcher, 2861 XZ Bergambacht, The Netherlands; remco.a.nederlof@gmail.com; 5 Animal Science Department, Biomedical Primate Research Centre, 2288 GJ Rijswijk, The Netherlands; bakker@bprc.nl

**Keywords:** reptiles, dystocia, egg-binding, reproductive disorder, hepatic lipidosis, treatment, diagnostic imaging, surgery

## Abstract

Dystocia represents a multifactorial and clinically significant reproductive disorder affecting a broad spectrum of reptilian species. Commonly resulting from prolonged vitellogenesis, endocrine disruption, or hepatic lipidosis, dystocia is often exacerbated by suboptimal husbandry or concurrent disease. This review critically evaluates the etiology, diagnostic criteria, and therapeutic interventions associated with this condition. Emphasis is placed on the interplay between metabolic exhaustion and hepatic compromise, which may lower the threshold for surgical intervention. The efficacy and limitations of oxytocin-based protocols are discussed in the context of hormonal receptor variability and response attenuation. Advanced diagnostic modalities, including ultrasonography, radiography/CT, and biochemical profiling, are reviewed for their utility in case stratification. Finally, surgical management options are considered for cases refractory to medical treatment, with attention paid to timing, anesthetic risk, and post-operative care. Collectively, this synthesis aims to inform evidence-based clinical decision-making and promote improved standards of care in reptile reproductive medicine.

## Simple Summary

Dystocia, or egg-binding, is a common reproductive emergency in reptiles, often arising from multifactorial systemic and environmental causes. This review focuses on the biochemical and hepatic determinants contributing to dystocia across major reptilian taxa, including lizards, snakes, and chelonians. Prolonged vitellogenesis, hepatic lipidosis, and metabolic disturbances such as hypocalcemia or elevated bile acids may impair oviductal contractility and egg passage, increasing the risk of reproductive failure. The pathophysiology of dystocia is examined with particular emphasis on liver function and its role in reproductive hormone metabolism, nutrient allocation, and systemic homeostasis. Diagnostic strategies, ranging from radiography and ultrasonography to plasma biochemistry, are evaluated for their relevance in assessing reproductive status and perioperative risk. Management options, including medical interventions (e.g., oxytocin protocols, supportive therapy) and surgical techniques (e.g., ovocentesis, coeliotomy, ovariosalpingectomy), are reviewed with consideration of anesthetic safety in metabolically compromised patients. This review aims to inform clinical decision-making by integrating diagnostic and therapeutic approaches with a strong emphasis on hepatic function and biochemical indicators, thereby improving outcomes in the veterinary management of reproductive failure in reptiles.

## 1. Introduction

Reproductive disorders are a significant clinical concern in captive reptiles, particularly as exotic species gain popularity in zoological collections, private ownership, and conservation programs. Dystocia is ranked as one of the most common and potentially life-threatening conditions found in this taxon [1]. Unlike domestic mammals, reptiles possess diverse reproductive strategies, influenced by phylogeny, environment, and ecology. These variations directly impact the presentation, progression, and management of reproductive diseases, such as dystocia. Despite its frequent occurrence, the pathophysiology of dystocia remains incompletely understood. Moreover, clinical management often varies significantly depending on the species, reproductive stage, and clinical and metabolic status of the animal. To address this disparity, a search was conducted for peer-reviewed articles in academic literature databases, such as PubMed, Scopus, and Web of Science, by using words and word combinations, such as dystocia, reptile, reproduction, reproductive disease, follicular stasis, hepatic lipidosis, diagnosis, surgery, and oxytocin, to identify potentially relevant publications. We then evaluated reports that we considered as clinically relevant, and articles that were deemed irrelevant to the review’s objectives were discarded. The scope of this review is to provide an integrated perspective on reptilian dystocia by examining the interplay between hormonal resistance, hepatic metabolic function, diagnostic strategies, and clinical management decisions, a multidimensional framework not previously synthesized in the published literature.

### 1.1. Reproductive Physiology Across Reptilian Taxa

Reptiles reproduce primarily through oviparity, although both viviparity and oviparous have evolved independently multiple times among squamates. Ovoviviparity is common in boas (Boidae), whereas true viviparity, with placental nutrient transfer, has been documented in several skinks (Scincidae) and in certain geckos such as *Naultinus* spp. (Gekkonidae) [2,3]. This reproductive diversity results in fundamental reproductive anatomical and physiological differences, which in turn influence the incidence and clinical presentation of reproductive disorders. Oviparous reptiles produce eggs that are retained within paired oviducts until deposition and may be either soft-shelled or hard-shelled, depending on the species’ environmental adaptations. In general, hard-shelled eggs are produced by species that nest in arid or semi-arid environments, while soft-shelled eggs are laid by those in moist habitats. These structural differences can affect the risk of specific complications, such as egg fracture or retention. In contrast, viviparous and oviparous species develop internal embryos, with the former exhibiting varying degrees of placentation and maternal nutrient transfer, and the latter relying primarily on yolk nutrients for embryonic development [4].

Reproductive endocrinology in reptiles is regulated by a tightly coordinated interaction of hypothalamic–pituitary–gonadal (HPG) axis hormones. The onset of vitellogenesis is driven by circulating estrogen, primarily estradiol-17β, which stimulates the hepatic production of yolk precursors and follicular development [5,6]. As follicles mature, progesterone concentrations increase. This rise initiates oviductal preparation for ovulation and shell formation. After ovulation, oviductal motility is primarily controlled by neurohypophyseal hormones, especially arginine vasotocin (AVT). Prostaglandins, including prostaglandin F2α (PGF2α), provide additional regulation. These mediators trigger calcium-dependent rhythmic contractions of the oviductal smooth muscle, enabling effective egg transport and oviposition [7].

Disruption in this hormonal cascade at any point, as well as lack of calcium, whether from environmental, nutritional, or pathological causes, can lead to follicular arrest, ovulatory failure, or oviductal dystocia. Hormonal resistance or dysfunction, particularly involving AVT receptors, underlie most cases of dystocia, especially in iguanas, chelonians, and skinks [8,9].

Anatomical and physiological differences among reptile taxa influence both the clinical presentation and diagnostic challenges of reproductive disorders. Chelonians possess a rigid pelvic canal that can obstruct oviposition, particularly in cases of pelvic deformities or oversized eggs [10]. Lizards, such as iguanas and agamids (Agamidae), have paired ovaries and oviducts that may ovulate asynchronously, sometimes resulting in the accumulation of yolk-filled follicles. While this has traditionally been referred to as pre-ovulatory follicular stasis (POFS), recent work suggests this condition may be overdiagnosed or misunderstood, with some cases representing normal physiological variation rather than true pathology [11]. Snakes have elongated coelomic cavities and linear reproductive tracts, which can obscure clinical detection of retained eggs until late in the disease process [12]. Crocodilians, though less commonly treated in clinical settings, display seasonal reproductive cycles and complex maternal behaviors, which may predispose them to oviposition failure under suboptimal captive conditions [13,14]. Finally, Dystocia is reported predominantly in captive reptiles, a pattern likely reflecting both captivity-associated risk factors (e.g., nutrition, husbandry, reproductive management) and substantial detection bias, as reproductive failure in free-ranging reptiles is rarely observed or documented; to the authors’ knowledges, no studies have directly compared dystocia frequency between captive and wild populations. Reliable estimates of dystocia frequency across major reptile groups (snakes, lizards, chelonians) are currently unavailable, as published data are largely species-specific and context-dependent (e.g., breeding colonies or retrospective clinical caseloads), precluding robust cross-taxonomic comparisons [15,16,17].

### 1.2. Dystocia and Related Reproductive Disorders

Dystocia in reptiles, also referred as egg-binding or ovostasis, is defined as a failure to successfully oviposit or give birth to viable offspring within a biologically appropriate timeframe. This may manifest either as POFS, where follicles fail to ovulate and persist within the coelomic cavity, or as post-ovulatory dystocia (POD), involving the retention of shelled eggs or fetuses within the oviduct. This condition can have functional, mechanical, environmental, or metabolic causes [1].

Reproductive disorders in reptiles are broadly categorized into pre-ovulatory and post-ovulatory pathologies, each with distinct pathophysiological mechanisms and clinical implications. POFS is characterized by the failure of mature ovarian follicles to ovulate, often resulting from chronic reproductive stimulation in the absence of appropriate environmental or social cues, or due to endocrine dysfunction. These retained follicles may persist, undergo fibrosis, degenerate into tumors, or become secondarily infected, potentially leading to systemic illness or yolk coelomitis if ruptured [18]. Follicular atresia, a normal physiological regression of non-ovulated follicles, must be distinguished from POFS, particularly in species like chameleons where ultrasonographic appearances may resemble pathological stasis. Misinterpretation of imaging may lead to unnecessary surgical or medical interventions, underscoring the need for context-specific clinical evaluation and a thorough understanding of species-specific reproductive physiology [19,20]. Discerning between these conditions often requires integrating imaging modalities (e.g., ultrasonography, radiography and computed tomography (CT)), hormone assays, and consideration of environmental and husbandry factors.

In contrast, POD involves the retention of ovulated and often fully shelled eggs or developed fetuses within the oviducts. Causes include physical obstruction, oviductal inertia, or inadequate environmental parameters necessary for nesting behavior, such as temperature, humidity, or substrate [21].

## 2. Integrative Scope: Hormonal Resistance, Hepatic Function, and Diagnostic Framework

Reproductive tract motility in reptiles is primarily regulated by AVT and prostaglandins, particularly PGF2α, with oxytocin often used empirically despite variable species sensitivity and poorly characterized receptor pharmacodynamics [6]. In cases of dystocia, particularly in iguanas, bearded dragons (*Pogona vitticeps*), and chelonians, prolonged retention of follicles or eggs may result in reduced responsiveness to AVT or oxytocin. This resistance is thought to arise from receptor downregulation, chronic smooth muscle distension, and/or local inflammation. This hormone-resistance renders medical therapy ineffective beyond a narrow therapeutic window [8,10].

Simultaneously, the liver plays a central role in reproductive physiology by synthesizing vitellogenin under estrogenic stimulation, but chronic vitellogenesis or reproductive stasis can induce hepatic lipidosis, hypoalbuminemia, and elevated liver enzymes, which are frequently reported in severe or chronic cases of POFS [22]. However, recent clinical reviews emphasize that these biochemical changes are often absent or only mild in early or functional cases; thus, normal blood chemistry does not reliably exclude POFS, and should not be used as a primary diagnostic criterion [11]. This is believed to reflect a negative energy balance associated with sustained metabolic demand, in which mobilization of peripheral energy reserves may contribute to hepatic triglyceride accumulation. Hypoalbuminemia may also develop in affected individuals, potentially due to decreased hepatic protein synthesis or the preferential diversion of amino acids toward vitellogenin production rather than albumin [23]. In contrast, hepatic changes appear to be significantly less prominent or relevant in POD. While POFS is often associated with prolonged estrogenic stimulation and sustained vitellogenesis, factors that predispose to hepatic lipidosis, hypoalbuminemia, and elevated liver enzymes, POD occurs after ovulation, when vitellogenic activity has ceased or markedly declined [24]. Consequently, the hepatic metabolic burden is presumed to be lower in POD. Available literature primarily describes POD in terms of mechanical obstruction, oviductal inertia, or egg-associated coelomitis, with little emphasis on hepatic involvement [1,25]. To date, no peer-reviewed studies have reported consistent alterations in liver enzymes or serum proteins specific to POD, suggesting that hepatic pathology, if present, is likely secondary to systemic inflammation or sepsis rather than a direct consequence of reproductive physiology. Further comparative studies would be beneficial to confirm this distinction. These alterations, along with elevated liver enzymes, are frequently observed in clinical cases of reproductive disorders, such as follicular stasis. Consequently, the presence of elevated liver enzymes or hepatic lipidosis may be a significant diagnostic indicator in POFS, but are rarely observed in other reproductive conditions such as POD [26,27]. This hepatic dysfunction not only compromises anesthetic safety but may also impair reproductive hormone metabolism, further perpetuating follicular arrest. These hepatic alterations, including elevated liver enzymes, are most frequently observed in reptiles with POFS, due to prolonged vitellogenesis and estrogenic stimulation. Histopathological and epidemiological studies in bearded dragons confirm that reproductively active females are significantly predisposed to hepatic lipid accumulation, with adult females showing substantially higher odds of moderate to severe hepatic lipidosis compared to males [28].

In hepatic lipidosis, fat-laden hepatocytes impair normal liver function, including the metabolism and clearance of estradiol-17β, normally processed through hydroxylation and conjugation pathways [29]. As a result, persistently elevated estradiol-17β levels drive continued hepatic vitellogenin synthesis and follicular development. In the absence of ovulation, this creates a self-reinforcing feedback loop: sustained estrogenic stimulation exacerbates hepatic lipidosis, which in turn further impairs hormone clearance and perpetuates follicular arrest [28,29].

As previously discussed, reproductive disorders such as follicular stasis and dystocia often result from a complex interplay between hepatic dysfunction and hormonal dysregulation. Understanding these mechanisms is essential for guiding diagnostic and therapeutic decision-making.

**Figure 1:**
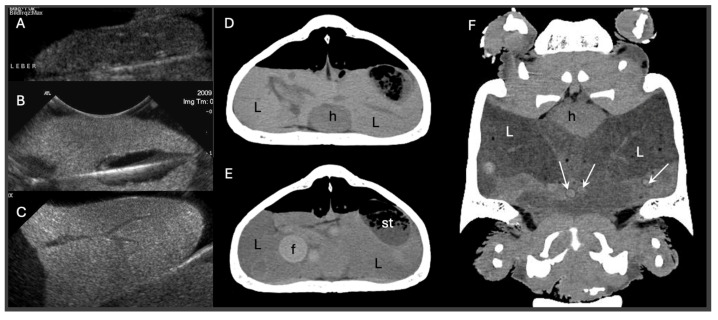
Diagnostic imaging of the liver in Hermann’s tortoises (*Testudo hermanni*) with and without hepatic lipidosis. (**A**–**C**) Ultrasonographic images obtained in a water bath in three different individuals. (**A**) Normal liver with homogeneous, finely granular parenchyma and moderate echogenicity. (**B**,**C**) Livers with hepatic lipidosis, showing diffusely increased echogenicity; in (**C**) the liver is also markedly enlarged with rounded margins. (**D**–**F**) CT images acquired in soft-tissue window. (**D**,**E**) Transverse images of the same female before the breeding season (**D**) and during folliculogenesis (**E**). The liver (L) is initially hyperdense to isodense relative to skeletal muscle and myocardium (h) with hypodense intrahepatic vessels, but becomes mildly to moderately hypodense during follicular development. A hyperdense, normal follicle (f) is visible in the right lateral coelomic cavity in (**E**), and the stomach (st) is on the left. (**F**) Dorsal plane reconstruction of a different tortoise with severe fatty liver disease and marked reduction in hepatic attenuation. The hepatic parenchyma contains multiple small fat-attenuating foci, and several small follicles (arrows) show a hypodense peripheral rim, suggestive of atresia. The described ultrasonographic and computed tomographic hepatic features are consistent with published imaging descriptions of normal liver anatomy and hepatic lipidosis in chelonians [33,35].

Diagnostic imaging plays a pivotal role in differentiating pre- from post-ovulatory reproductive disease. Ultrasonography is particularly useful for evaluating follicular integrity, echogenicity, inner architecture and vascularization, especially in lizards and snakes, while radiography allows for the evaluation of egg calcification. CT is the more powerful imaging tool in chelonians, as it can display follicles, eggs and salpinx as well as the skeleton. Position, number, size, shape, density and inner architecture can be differentiated as well as causes of soft tissue distension, mechanical obstruction or other comorbidities [30,31]. Furthermore, the density of the liver may be indicative for hepatic lipidosis in all reptiles [32,33,34] (Figure 1).

Reptile reproductive disorders are frequently complicated by concurrent hepatic dysfunction, yet standardized protocols for assessing hormonal responsiveness or determining optimal surgical timing remain lacking. Clinicians must therefore rely on a combination of biochemical, imaging, and clinical indicators, each with its own limitations. Traditional liver biochemical markers, including aspartate aminotransferase (AST), bile acids, and albumin, provide useful context but often remain within normal limits even in animals with histologically confirmed hepatic lipidosis, limiting their diagnostic reliability [27,36]. Imaging has emerged as a more sensitive tool in this setting. Unenhanced CT allows for quantitative assessment of hepatic attenuation, which correlates strongly with lipid infiltration and clearly outperforms ultrasonography and serum biochemistry in sensitivity. These findings underscore the need for an integrated diagnostic framework. Incorporating CT-based liver density evaluation alongside reproductive imaging and interpretation of clinical timing offers a more accurate basis for risk stratification and therapeutic planning. This chapter therefore focuses on bridging this diagnostic gap by outlining a multifaceted, evidence-based approach to evaluating reproductive disorders in reptiles with potential hepatic involvement.

## 3. Hormonal, Environmental, and Species-Specific Drivers

Dystocia in reptiles arises from a complex interplay of physiological, environmental, nutritional, and anatomical factors. Despite the frequent presentation of dystocia in captive reptile medicine, most proposed risk factors remain extrapolated from case experience rather than experimental confirmation. This section reframes the etiology of dystocia under three key clinical dimensions: endocrine–metabolic dysfunction, environmental and husbandry mismatch, and species-specific anatomical and physiological predispositions.

### 3.1. Endocrine-Metabolic Dysfunction and Reproductive Arrest

Reproductive arrest in reptiles, encompassing both POFS and POD, is frequently the result of underlying endocrine or metabolic dysfunctions. These disruptions are often subclinical until reproductive failure occurs, and they play a particularly critical role in oviparous lizards, snakes, and chelonians maintained in captivity. While reproductive endocrinology in reptiles shares hormonal analogs with other vertebrates, the nuances of estrogen-driven vitellogenesis, progesterone-dependent oviductal preparation, and AVT-mediated oviposition are finely tuned by environmental and physiological cues—disruptions of which can precipitate ovulatory failure or impaired egg passage [7,37].

Estradiol-17β is the primary estrogen responsible for inducing hepatic vitellogenin synthesis in reptiles, as demonstrated in Kemp’s ridley sea turtles (*Lepidochelys kempii*), where experimental administration of estradiol-17β resulted in a marked increase in plasma vitellogenin concentrations within one week [38]. Chronic elevation, often due to prolonged exposure to reproductive triggers in the absence of mating or oviposition cues, can cause excessive hepatic lipogenesis. This may manifest clinically as hepatic lipidosis, which can interfere with hepatic detoxification, albumin synthesis, and hormone clearance [18,39]. For example, in Fiji island banded iguanas (*Brachylophus fasciatus*), repeated reproductive cycling without oviposition, which commonly occurs in single-housed females, leads to coelomic accumulation of yolk-laden follicles and progressive hepatic degeneration. In the majority of cases, yolk leakage was linked to the presence of large vitellogenic follicles undergoing atresia, which subsequently induced yolk coelomitis, characterized by pronounced mesothelial proliferation [39]. A histopathological study in bearded dragons showed that reproductive females had a significantly higher prevalence of hepatic lipid infiltration, with advanced cases correlating with inactive ovaries and non-responsive dystocia [28]. This hepatic compromise often coexists with hypoalbuminemia and impaired coagulation, posing anesthetic risks if surgical intervention is later required. Additionally, hepatic impairment alters estrogen and progesterone metabolism, potentially contributing to a cycle of continued follicular development without ovulation, particularly in species with high hepatic lipid loads. For instance, in the North African spiny-tailed lizard (*Uromastyx acanthinura*), the estrogen receptor beta (ERβ) is predominantly involved in this regulatory mechanism and is associated with the inhibition of vitellogenin synthesis during certain reproductive phases [40]. Although estrogen, particularly estradiol-17β, generally promotes vitellogenesis via estrogen receptor alpha (ERα), ERβ may play an inhibitory or modulatory role, potentially acting to fine-tune or suppress estrogenic activity under specific physiological conditions. Therefore, disruption in liver function could impair this estrogen-mediated pathway, resulting in reduced vitellogenin production and compromised reproductive capacity. Progesterone often acts to counterbalance estrogen’s effects on vitellogenesis. The presence of hepatic progesterone receptors (PRA and PRB) in *Uromastyx acanthinura*, and their association with inhibition of vitellogenin synthesis during specific reproductive phases, presents an intriguing avenue for potential therapeutic intervention [40]. This mechanism suggests that progesterone or selective progesterone receptor modulators could theoretically be explored to downregulate vitellogenesis in cases of chronic reproductive stimulation, such as POFS, where prolonged estrogenic stimulation and failure of ovulation result in persistent vitellogenesis and follicular retention [29,38]. However, this approach would require caution and species-specific validation, as the hormonal feedback loops governing reproduction are complex and poorly understood in many reptilian taxa. Further research into receptor distribution, ligand specificity, and endocrine timing across species is needed before clinical application could be considered.

Reproductive success in reptiles is calcium-dependent, particularly during egg calcification and oviductal contraction. Hypocalcemia may result from inadequate dietary intake, poor gastrointestinal absorption, or insufficient ultraviolet B (UVB) exposure leading to reduced vitamin D_3_ (cholecalciferol) synthesis. Additionally, repeated reproductive cycling, such as in females housed with males year-round, can lead to chronically increased calcium demand and depletion of physiological calcium stores, further predisposing individuals to hypocalcemia [41]. Calcium deficiency contributes to uterine inertia, especially in chelonians and gravid lizards, where sustained oviductal contraction is essential for successful oviposition [42]. In red-eared sliders (*Trachemys scripta elegans*), egg retention has been directly associated with ionized calcium levels below physiological thresholds, necessitating injectable calcium and oxytocin to restore oviductal motility [6].

Finally, yolk coelomitis, a severe inflammatory sequela of reproductive dysfunction, may develop secondary to chronic follicular atresia and vitellogenic failure. This condition, while often classified under reproductive or infectious pathology, is closely tied to endocrine-metabolic dysfunction, particularly when associated with hepatic impairment, prolonged vitellogenesis, or immune suppression. In one reported case, a captive white-throated monitor lizard (*Varanus albigularis*) exhibited nonspecific clinical signs including lethargy, anorexia, and coelomic distension before succumbing to severe coelomitis. Post-mortem findings revealed florid mesothelial hyperplasia attributed to yolk leakage from atretic follicles, underscoring the systemic impact of prolonged endocrine and metabolic dysregulation [43].

While hepatic lipidosis and hypocalcemia are widely recognized contributors to reproductive arrest in reptiles, there remains a significant gap in understanding the underlying endocrine and metabolic mechanisms. Notably, a limited number of studies have examined the expression of AVT receptors in oviductal smooth muscle, a factor that could influence oviductal contractility and contribute to dystocia. Additionally, comprehensive histopathological analyses of the liver in reptiles with idiopathic POFS or recurrent reproductive failure are lacking, despite evidence suggesting a link between hepatic lipid accumulation and reproductive disorders. Furthermore, comparative studies on calcium metabolism under varying UVB exposure and dietary regimes across different reptile taxa are sparse, yet such factors are crucial for proper reproductive function. To address these gaps, future research should adopt a systems biology approach, integrating assessments of hormonal levels, receptor functionality, liver function, and immune responses. This holistic perspective will enhance our understanding of the thresholds at which physiological variations transition into pathological conditions, ultimately improving diagnostic and therapeutic strategies for reproductive disorders in reptiles.

### 3.2. Risk Factors

#### 3.2.1. Exogenous Factors

Environmental parameters play a critical role in reproductive stasis in reptiles; inadequate temperature gradients and humidity levels can disrupt normal oviductal contractility and nesting behavior, leading to egg retention. For instance, in chelonians, poor temperature regulation and improper humidity have been recognized as key contributors to reproductive disorders, including dystocia and follicular stasis [44]. The absence of appropriate nesting substrates or sites can cause females to retain eggs, as the lack of suitable laying environments impedes the natural oviposition process [45]. Nutritional deficiencies, particularly hypocalcemia resulting from inadequate dietary calcium or vitamin D3, are significant contributors to dystocia. Hypocalcemia impairs muscle contractions necessary for egg laying, leading to uterine inertia. In lizards, hypocalcemia has been associated with clinical signs such as muscle tremors and lethargy, which are commonly observed in cases of dystocia [45]. Additionally, metabolic bone disease, meaning nutritional secondary hyperparathyroidism, resulting from chronic calcium and vitamin D3 deficiencies, has been reported in up to 84.4% of captive lizards in certain studies, indicating a widespread issue that can predispose reptiles to reproductive complications [46].

#### 3.2.2. Endogenous Factors

Endocrine dysfunctions also play a role; disruptions in the hypothalamic–pituitary–gonadal axis can lead to persistent follicular growth without ovulation, culminating in POFS [47]. While specific studies quantifying this in reptiles are limited, the association between hormonal imbalances and reproductive issues is well-documented in veterinary literature [48]. Any space-occupying process within the coelomic cavity, as well as infections such as salpingitis and oophoritis, neoplasia, or urolithiasis, directly obstruct egg passage, or may lead to inflammation and adhesions that obstruct egg passage, particularly in species like tortoises and iguanids [25]. Physiological factors, including age and reproductive history, influence the risk of dystocia. Primiparous females and older reptiles are more susceptible, potentially due to inexperience or reduced muscular efficiency [49]. In addition, the effects of nutritional secondary hyperparathyroidism may compound with age, particularly in animals housed in suboptimal environments, where cumulative depletion of calcium and other resources further impairs the ability to pass eggs effectively [50]. Obesity, often resulting from overfeeding in captivity, can lead to excessive fat deposits in the coelomic cavity, physically impeding egg passage and increasing the risk of dystocia [45]. Moreover, once such animals enter a negative energy balance, during anorexia, reproductive stasis, or illness, they mobilize these fat stores, predisposing them to hepatic lipidosis [28].

### 3.3. Species-Specific Tendencies

While general pathophysiological mechanisms underlying dystocia in reptiles are often shared, the clinical presentation, progression, and optimal management of these reproductive disorders vary significantly between taxonomic groups. This variation is rooted in species-specific anatomical structures, reproductive physiology, environmental needs, and behavioral traits. For this reason, identifying and understanding taxon-specific predispositions is crucial for accurate diagnosis, timely intervention, and effective treatment planning. The most common types of dystocia, relevant species, risk factors, and supporting literature are shown for chelonians in Table 1, for snakes in Table 2, and for lizards in Table 3. By presenting these distinctions in a structured format, this review emphasizes the importance of tailored clinical approaches based on species, rather than applying uniform protocols across all reptiles, a practice that may result in misdiagnosis or suboptimal outcomes.

## 4. Diagnostic Workup

Dystocia often presents with nonspecific clinical signs such as anorexia, lethargy, coelomic distension, and restlessness. In some cases, straining behavior or cloacal prolapse may be observed. These signs can persist for weeks or even months, making early detection challenging [44,59].

### 4.1. Radiographic Examination

Radiography remains one of the most accessible and widely used diagnostic tools in the assessment of dystocia in reptiles. It allows for non-invasive visualization of mineralized reproductive material, such as shelled eggs, dystrophic calcifications, as well as gas-filled coelomic structures, that may be suggestive for necrosis or secondary infection.

Standard lateral and dorsoventral radiographic views are typically sufficient for most lizard and chelonian species. In snakes, coiling often complicates interpretation, so careful straightening and multiple views may be needed [60]. For chelonians, horizontal beam radiography is highly valuable, particularly when using cranio-caudal or oblique projections to assess the pelvic inlet and caudal oviduct [61]. In gravid reptiles, a normal radiograph shows uniformly round to ovoid, more or less mineralized (depending on the species) eggs arranged linearly or clustered in the coelomic cavity. The number of eggs can be counted and the size measured, if sufficiently mineralized, which assists in determining normal versus pathological retention [1]. In lizards and snakes with obstructive dystocia, eggs may be crowded cranial to a narrowed pelvic region or appear deformed due to prolonged retention and compression. For example, a report in a bearded dragon describes the presence of three retained eggs, which caused caudal oviductal dilation and cloacal distension [56].

**Figure 2:**
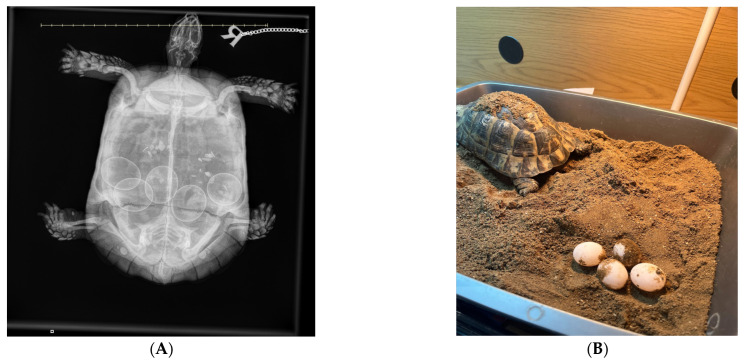
(**A**) Dorsoventral radiographic view of five retained eggs in a female Moroccan tortoise (*Testudo graeca marokkensis*). (**B**) Oviposition in a captive female *Testudo graeca marokensis* housed in an indoor enclosure with a prepared sand substrate, after medical management with injections of calcium gluconate, oxytocin and morphine.

**Figure 3:**
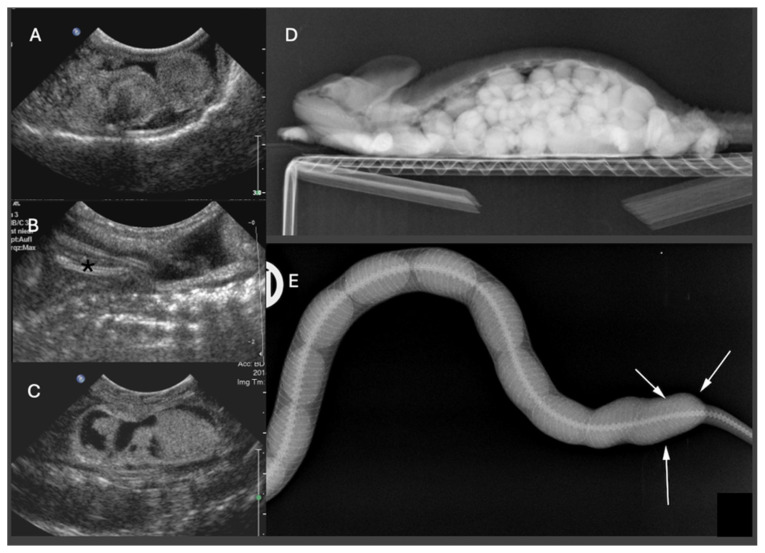
Diagnostic imaging of reproductive pathology in reptiles. (**A**) Ultrasonographic image of multiple mildly irregular, heterogeneous follicles with a small amount of surrounding free fluid in a bearded dragon; no interval change was noted over ten days despite clinical deterioration, consistent with follicular stasis. (**B**) Ultrasonographic evaluation of a gravid garter snake (*Thamnophis sirtalis*); the skeleton of a fetus (*) is visible, but no cardiac activity was detected, confirming fetal death. (**C**) Serial ultrasonography of a boa showing progressive degeneration of several eggs over weeks, including loss of internal architecture and non-viable fetal structures. (**D**) Lateral radiograph of a veiled chameleon (*Chamaeleo calyptratus*) with severe superovulation; the lungs are markedly compressed and there is profound skeletal demineralization associated with chronic reproductive disease. (**E**) Dorsoventral radiograph of a corn snake (*Pantherophis guttatus*) with an enlarged, obstructed egg at the level of the cloaca (arrows), tightly apposed to a second egg.

Radiographic examination can reveal several abnormalities indicative of dystocia in reptiles (Figure 2). One notable finding is the presence of overlapping or collapsed eggs, which typically suggest mechanical obstruction or repeated failed oviposition attempts (Figure 3). These eggs may appear irregular or crumpled on radiographs and are frequently observed in calcium-deficient individuals, particularly in green iguanas (*Iguana iguana*) [56]. Another diagnostic feature includes the detection of mineralized follicular shadows lacking definitive shell structure. While these may be misinterpreted as early-stage eggs, they more likely represent dystrophic calcifications associated with chronic follicular stasis or coelomitis [25]. In oviparous snakes such as ball pythons (*Python regius*), egg retention beyond the expected gestation period, typically 45 to 60 days post-ovulation, combined with radiographic evidence of persistent, calcified eggs, has been considered diagnostic for dystocia [62]. The presence of free intra-coelomic gas or gas within an egg on radiographs is another critical finding, the former being often indicative of oviductal rupture, secondary infection, or necrosis [63]. Additionally, skeletal abnormalities, particularly affecting the spine or pelvis, can predispose animals to obstructive dystocia. In chelonian species, such as spur-thighed tortoises (*Testudo graeca*), congenital deformities or narrowed pelvic outlets may be radiographically detectable and should be considered during reproductive evaluations [25].

### 4.2. Ultrasonography

**Figure 4:**
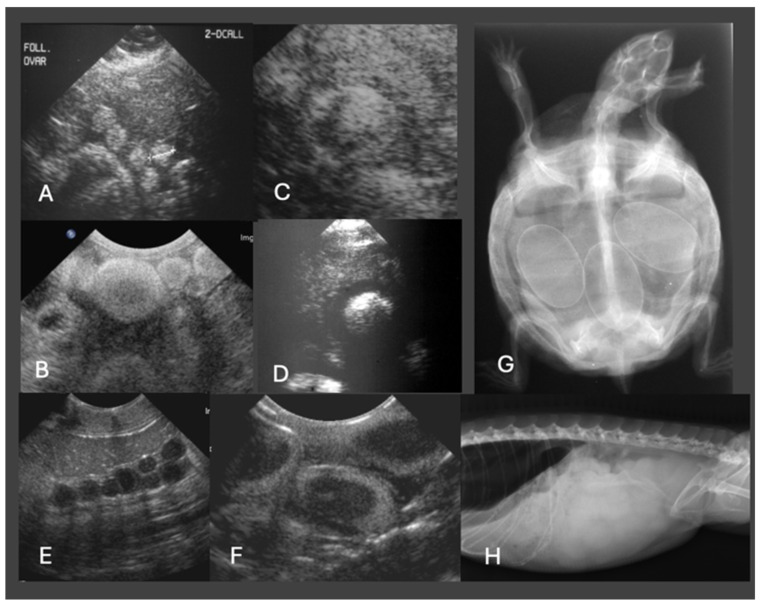
Diagnostic imaging of normal follicles and eggs in chelonians and squamates. (**A**–**C**,**E**,**F**,**H**) Ultrasonographic appearances of normal ovarian follicles and oviductal eggs in Hermann’s tortoises (**A**–**D**), a box turtle (**G**), a python (**E**), a water dragon (*Intellagama lesueurii*) (**F**), and a green iguana (**H**). (**A**) Primary follicles with a homogeneous, mildly hyperechoic appearance typical of chelonians. (**B**) Mature, moderately sized hyperechoic follicles with characteristic distal acoustic shadowing. (**C**) Heterogeneous echotexture within a small follicle, consistent with early atresia. (**D**) Ovulated follicle containing yolk with a peripheral hypoechoic rim (albumen); a faint outer hyperechoic line suggests early eggshell mineralization. (**E**) Primary follicles in squamates (python) are comparatively hypoechoic. (**F**) Normal elongated oval eggs with soft shells in a water dragon. (**G**) Dorsoventral radiograph of a box turtle showing a normal clutch of large, uniformly sized eggs without evidence of dystocia. (**H**) Lateral radiograph of a green iguana displaying multiple ovoid soft-tissue-dense eggs deposited two days earlier.

Ultrasonography is the modality of choice for assessing POFS, soft tissue changes in the oviducts, and associated coelomic pathology, especially in lizards and snakes. Ultrasonography can be performed in dorsal or lateral recumbency or even upright position using a high-frequency linear transducer. For chelonians, the prefemoral fossa provides an acoustic window to the reproductive tract, but effective visualization depends on the size of animal and the shape of the transducer. Usually, adults have well-developed fossae, while in smaller or younger individuals, limited soft tissue access may hinder probe placement and diagnostic clarity [10,64]. Developing follicles show a homogenous, round, anechoic appearance, and may be arranged in clusters. Only in chelonians do already small follicles appear hyperechoic. Normal oviducts should not be distended or surrounded by hyperechoic fat or fluid. In gravid females, normal oviductal eggs appear as oval anechoic to mildly hypoechoic structures with a thin hyperechoic wall. As calcification progresses, distal acoustic shadowing may begin to appear [61,65] (Figure 4).

Recent work on female veiled chameleons (*Chamaeleo calyptratus*) has demonstrated that follicular atresia is a more dynamic and diagnostically complex process than previously assumed. In a longitudinal imaging study using ultrasonography and CT, four distinct stages of follicular development, previtellogenesis, vitellogenesis, gravidity, and atresia, were identified, and subsequently confirmed by histology. Atretic follicles were frequently present in multiple generations within the same animal, often without apparent disruption of normal folliculogenesis, suggesting that atresia can be a physiological process across several cycles. However, this diagnostic nuance presents clinical challenges: echogenic changes seen during ultrasonography (e.g., heterogeneous content and irregular follicular shape) can mimic POFS, potentially leading to overtreatment, including unnecessary surgical or hormonal interventions [49].

**Figure 5:**
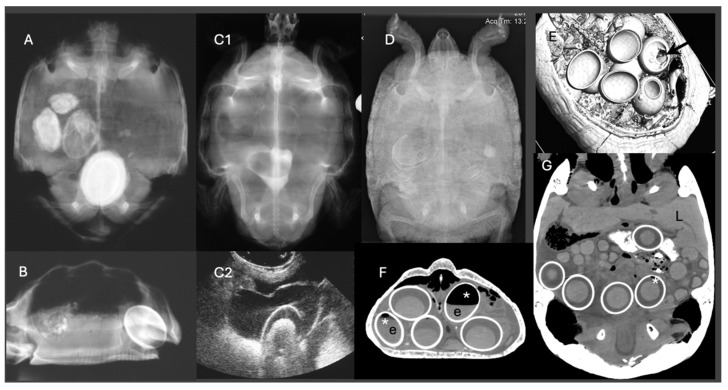
Diagnostic imaging of dystocia in chelonians. (**A**) Dorsoventral radiograph of a Hermann’s tortoise with chronic dystocia; one oversized egg is obstructed at the pelvic canal, with marked secondary shell mineralization. Additional smaller eggs with irregular shells are present. (**B**) Lateral radiograph of a spider tortoise showing an egg trapped at the cloacal opening due to a caudal shell aperture that is too narrow for passage; the cranial pole of the shell is notably thicker than the caudal pole already positioned near the vent. (**C1**) Dorsoventral radiograph of a red-eared slider turtle after cloacal administration of contrast medium, demonstrating an egg located within the urinary bladder; (**C2**) ultrasound confirmed its intravesical position. (**D**) Dorsoventral radiograph of a Hermann’s tortoise with metabolic bone disease and multiple fractured eggs. (**E**) Three-dimensional CT model of a Hermann’s tortoise with the carapace and eggs virtually removed; one egg near the right inguinal window (arrow) shows an impression fracture, likely caused by vigorous palpation. (**F**,**G**) Transverse and coronal CT images (soft-tissue window) of two Hermann’s tortoises. Several eggs contain gas (*) and exhibit loss of internal architecture (e), consistent with decomposition. Follicles of varying size are visible, some with abnormal contour or a peripheral hypodense rim suggestive of degeneration or stasis. In (**G**), the liver (L) displays normal hyperdense parenchyma.

In cases of POFS, retained follicles remain within the ovary without evidence of ovulation. Ultrasonographically, these structures appear as enlarged, rounded, anechoic or heterogeneously hypoechoic areas, often devoid of peripheral vascular flow on Doppler examination [65] (Figure 5). In veiled chameleons, follicles exceeding 10 mm in diameter and persisting for several weeks without regression are considered diagnostic for POFS [55]. In more chronic cases of POFS or reproductive failure, follicular regression or atresia may result in internal echoes, septations, or hyperechoic rims, which can mimic abscesses or neoplastic masses. Mineralization of retained follicles has also been reported in chronic or advanced stasis [28]. Finally, Doppler ultrasonography may aid in differentiating pathologies: the absence of peripheral vascular flow supports a diagnosis of inactive or atretic follicles, while hypervascularization may suggest inflammation, infection, or neoplasia [65].

POD is characterized ultrasonographically by oviductal eggs that may appear irregularly shaped, collapsed, or surrounded by echogenic coelomic fluid, among other features. In a reported case in a bearded dragon, ultrasonography revealed free coelomic fluid and distorted oviductal structures encasing abnormally positioned eggs, consistent with dystocia complicated by yolk coelomitis [56]. In more severe presentations, oviductal distension or impaction may be evident, often manifesting as tubular, fluid-filled structures with echogenic debris. The presence of gas suggests secondary bacterial infection or necrosis, while mural thickening and loss of the normal oviductal wall layering may also be visualized [66,67].

### 4.3. Computed Tomography

CT has emerged as a powerful diagnostic modality in the assessment of reptile dystocia, offering high resolution and three-dimensional visualization of mineralized and soft tissue structures. In chelonians, even very small ovarian follicles can usually be identified on non-contrast CT, whereas in smaller lizards intravenous (IV) contrast medium is often required to reliably delineate ovarian and oviductal soft tissues. For further evaluation of vascular changes in the oviductal or ovarian tissues contrast medium application is needed [61]. CT allows for precise evaluation of the number, shape, and positioning of eggs or follicles, which is particularly useful in species with complex internal anatomy such as snakes or in chelonians being enclosed in a bony shell. It also overcomes the limitations posed by complex external anatomy, like the bony shells of chelonians and osteodermal plates of crocodilians, that often obscure or distort conventional imaging modalities [61].

For example, in a Greek tortoise, CT imaging was used to identify a congenital pelvic narrowing that contributed to recurrent dystocia [44]. CT also allows for clear differentiation between shelled eggs, calcified follicles, and inflammatory or necrotic debris based on tissue density, which is essential in chronic or complicated cases [61]. CT was successfully used in ball python (*Python regius*) to identify malformed and obstructively positioned post-ovulatory eggs causing oviductal dilation [10]. Furthermore, CT aids in detecting secondary complications, such as coelomic gas, fluid, or oviductal rupture. It also plays a critical role in pre-surgical planning by delineating anatomical landmarks. Although the technique may require anesthesia in for example agile individuals and requires access to specialized equipment, its diagnostic precision makes it an invaluable tool in the evaluation of suspected dystocia cases in reptiles. Although MRI has not yet been widely studied in reptilian reproductive medicine, it holds theoretical promise for superior soft tissue contrast, especially in visualizing vascularization, oviductal edema, or early-stage follicular structures, suggesting a potential future role alongside ultrasound and CT [68]. However, it usually requires general anesthesia and takes much longer than CT.

### 4.4. Endoscopy

Endoscopic evaluation, particularly cloacoscopy and coelioscopy, constitutes a minimally invasive and highly informative diagnostic approach in cases of reptile dystocia. Cloacoscopy is especially useful in cases of POD to directly visualize retained eggs in the caudal oviduct or cloaca, and to assess mucosal health, obstructions, or signs of infection. This technique allows for targeted flushing, biopsy, or even assisted egg removal without the need for full surgical intervention [59].

Coelioscopy is typically performed via the prefemoral approach in lizards and chelonians, and allows for direct inspection of ovarian follicles, oviducts, and associated structures within the coelomic cavity [69]. For example, in a case series of female bearded dragons with suspected POFS, coelioscopy enabled clear visualization of enlarged, regressing follicles and oviductal abnormalities, which guided decision-making for salpingectomy [70]. Endoscopy also allows for dynamic assessment (e.g., identifying adhesions, inflammation, or coelomic effusion) and can be used therapeutically for follicular aspiration or lavage. While endoscope-assisted coeliotomy requires specialized training and equipment, it can enhance diagnostic accuracy and reduce invasiveness in select cases compared to coelioscopic plastrotomy. However, in chelonians, particularly tortoises, the endoscopic approach often demands longer operative time with no clear wound-healing advantage. Moreover, anatomical constraints, e.g., shell morphology, frequently limit access [71]. In chelonians, an important additional consideration is the occurrence of ectopic eggs in the urinary bladder. Such misdirected eggs can arise acutely during dystocia or present later as mineralized uroliths, with potential for chronic irritation, hematuria, or lower urinary tract dysfunction. Radiography (sometimes with retrograde contrast media application), CT and even sonography may be used to detect these mineralized structures. However, cystoscopy may be the preferred tool, enabling direct visualization and minimally invasive retrieval with snares or baskets [72,73]. Recognition of this phenomenon is vital: retention of ectopic eggs can complicate medical treatment of dystocia, obscure interpretation of imaging and delaying required intervention, thus bridging the diagnostic pathways of dystocia and urolithiasis.

### 4.5. Hematology

Comprehensive blood analyses, including hematology and biochemistry, are vital for diagnosis and management. Common hematologic findings in reptiles with dystocia include heterophilia, anemia, and monocytosis, consistent with chronic inflammation or stress responses. For instance, in Greek tortoises, follicular stasis has been associated with anemia and leukopenia. Heterophilia and monocytosis are observed in cases of ovarian teratomas and chronic egg retention, reflecting inflammatory responses [60]. In female common chameleons (*Chamaeleo chamaeleon*) with dystocia, hematologic evaluation revealed significant alterations compared with healthy post-reproductive controls, including increased heterophil and monocyte counts, consistent with inflammatory and stress-related responses [74]. However, interpreting anemia in reptiles requires caution, as venipuncture techniques often dilute samples with lymphatic fluid, particularly when using tail or subcarapacial sinuses, resulting in artifactually low PCV, RBC, and protein values [75]. Dystocic chameleons showed increased monocyte percentages and elevated AST levels, indicating tissue trauma associated with egg retention [74]. Finally, in African rock pythons (*Python sebae*), studies have reported variations in white blood cell counts, with elevated heterophils and lymphocytes in individuals with reproductive disorders, although specific values vary [76].

### 4.6. Blood Biochemistry

Metabolic disturbances, including alterations in blood glucose, are not uncommon in reproductive disease in reptiles. Hypoglycemia may be observed in pathological states such as egg yolk coelomitis [74]. However, it is important to recognize that some species, such as the common chameleon (*Chamaeleo chamaeleon*), exhibit higher baseline glucose levels, with mean values around 362 mg/dL, compared to the typical reptilian reference range of 60–100 mg/dL [74]. Increased AST activity, a marker of hepatic or muscular injury, was significantly elevated in dystocic chameleons compared to healthy controls. The frequent concurrent observation of hepatic lipidosis in these cases provides further support for a potential link between hepatic dysfunction and reproductive complications such as dystocia. Elevated creatine phosphokinase (CPK) levels may indicate skeletal muscle damage associated with prolonged egg retention. It is important to emphasize that it remains unclear whether these values exceeded established reference ranges, which are often broad or derived from limited sample sizes in exotic species. This underscores the challenge of interpreting clinicopathologic changes in reptiles, particularly when population-based reference intervals are poorly defined. When assessed alongside AST, CPK can assist clinicians in distinguishing between muscular and hepatic sources of AST elevation, an important diagnostic consideration in reptiles presenting with dystocia. A disproportionately elevated AST with normal or only mildly increased CPK may suggest hepatic involvement, whereas concurrent increases in both enzymes may reflect muscle injury, which could result from coelomic straining due to dystocia, but may also arise from other causes such as trauma, handling, prolonged recumbency, or systemic illness [77]. Hypercalcemia and hyperphosphatemia are common during vitellogenesis. However, in cases of chronic egg retention, total calcium and ionized calcium levels may decrease due to continuous deposition on retained eggs [44].

Hepatic dysfunction is a key concern in reptiles presenting with reproductive arrest, particularly those experiencing prolonged follicular stasis or anorexia. Among the hepatic pathologies associated with reproductive disorders, hepatic lipidosis is most frequently reported and can substantially alter plasma biochemical profiles. Elevated AST is a common indicator of hepatocellular injury. In dystocic bearded dragons, AST concentrations have been documented between 180 and 520 international units (IU)/Liter (reference range: 50–250 IU/L), with some individuals presenting values still within the upper end of the normal range. While elevated AST may serve as an indicator of underlying hepatic or muscular compromise, values between 180 and 250 IU/L overlap with reference intervals and thus should not be interpreted in isolation. Given the variability and non-specificity of AST in reptiles, it is best regarded as a supportive finding rather than a definitive diagnostic marker [28,78]. Plasma bile acids have also proven to be sensitive markers of liver dysfunction in reptiles. In chelonians, fasting and postprandial serum bile acid concentrations have been characterized in healthy red-eared terrapins (*Trachemys scripta elegans*), with consistently low fasting values and a significant postprandial increase following feeding, reflecting normal hepatobiliary function and enterohepatic circulation [79]. In the context of reproductive arrest and dystocia, these data provide an important physiological baseline, as prolonged anorexia, follicular stasis, or chronic vitellogenesis may blunt postprandial bile acid responses or mask early hepatobiliary dysfunction, thereby limiting the sensitivity of bile acids as diagnostic markers in early or functional reproductive disease. In green iguanas diagnosed with chronic hepatopathies, histologically confirmed as hepatic lipidosis, cirrhosis, hepatocellular carcinoma, fibrosis, and hepatocellular degeneration, plasma bile acid levels averaged 84.85 ± 22.29 μmol/L after 48 h of fasting, compared with 9.56 ± 8.52 μmol/L in healthy, fasted controls [80]. This finding confirms that various chronic liver diseases, including hepatic lipidosis, are associated with markedly elevated bile acids in green iguanas. However, it is important to note that such elevations were documented in end-stage liver disease; in subclinical cases such as vitellogenesis-induced hepatolipidosis, bile acid levels may remain within normal limits or even fall below detectable thresholds, limiting their diagnostic reliability in early reproductive hepatopathies. In green iguanas, plasma protein electrophoresis has established albumin concentrations in clinically healthy animals typically ranging from approximately 9–20 g/L, with significant variation related to season and physiological status. Values below these established baselines may therefore indicate pathological processes, and hypoalbuminemia in reptiles has been associated with chronic hepatic dysfunction, impaired protein synthesis, and inadequate dietary protein intake, particularly in the context of chronic disease [81,82]. Other differentials, such renal disease associated with protein loss should also be considered [83,84]. Accurate assessment of albumin concentrations in reptiles is method-dependent; photometric analyzers such as VetScan often underestimate true albumin levels due to protein-binding artifacts, and reliable quantification ideally requires gel electrophoresis, an approach that may be impractical in smaller species where sample volumes are limited. Additionally, while elevated uric acid levels are not specific to hepatic dysfunction, they may increase in cases of systemic illness or renal compromise. However, specific uric acid trends in dystocic individuals remain insufficiently characterized in the current literature.

Recent advances in reptile metabolomics have led to the identification of promising plasma biomarkers that can aid in the early diagnosis and monitoring of hepatic lipidosis. [85].

Β-hydroxybutyric acid (βHBA) is a ketone body involved in lipid metabolism and energy regulation during periods of anorexia or increased metabolic demand, such as folliculogenesis. In bearded dragons, significantly reduced levels of βHBA have been strongly associated with hepatic lipidosis. A plasma βHBA concentration below 272 µmol/L demonstrated a sensitivity of 86% and specificity of 100% for detecting moderate to severe hepatic lipidosis [27]. The suppression of ketogenesis, reflected in low βHBA, may indicate an overwhelmed or dysfunctional liver unable to compensate metabolically. Thus, βHBA serves as an early warning sign of metabolic exhaustion and hepatic burden in dystocic reptiles [86]. A recent study established reference intervals for βHBA in healthy adult central bearded dragons and evaluated a point-of-care ketone meter against a reference laboratory analyzer. The point-of-care device consistently underestimated BHBA concentrations compared with the laboratory method, showing a proportional negative bias. However, the total observed error remained within clinically acceptable limits, indicating that the device is still appropriate for routine clinical monitoring. The authors proposed a simple correction factor (multiplying point-of-care results by 0.9) to improve agreement between methods. Importantly, no significant interference was detected from hematocrit, lipemia, hemolysis, or blood glucose. This study also provided the first species-specific βHBA reference intervals for *Pogona vitticeps*, supporting the use of βHBA as an accessible metabolic biomarker in the assessment of hepatic lipid metabolism in lizards [87].

Succinic acid, a key intermediate in the tricarboxylic acid cycle, has been identified as a promising biomarker for hepatic lipidosis in bearded dragons. In a recent metabolomics study, plasma succinic acid concentrations exceeding 13.7 µmol/L were significantly associated with moderate to severe hepatic lipid infiltration. At this threshold, succinic acid demonstrated a sensitivity of 86% and a specificity of 100% for detecting moderate to severe lipidosis, with an area under the receiver operating characteristic curve of 0.98 [86]. In reproductive females, increased succinic acid may indicate mitochondrial dysfunction or altered energy metabolism, which could further compromise oocyte maturation, vitellogenesis, or uterine contractions. This makes succinate not only a marker of hepatic stress, but also a potential marker for impaired reproductive physiology [88].

Differentiating between metabolic changes due to fasting and true hepatic lipidosis presents a key diagnostic challenge in dystocic reptiles. Prolonged anorexia is common in females with follicular stasis or egg retention, leading to fat mobilization and altered levels of plasma metabolites such as βHBA and succinic acid. However, these changes may occur in both adaptive fasting states and pathological hepatic lipidosis, complicating interpretation [27]. However, these changes may occur in both adaptive fasting states and pathological hepatic lipidosis, complicating interpretation. In such cases, liver cytology via fine-needle aspiration may aid in diagnosis, as hepatocellular vacuolation, lipid-laden hepatocytes, and the presence of inflammatory cells can help distinguish true hepatic lipidosis from fasting-induced hepatic metabolic shift [28,89]. Nevertheless, hepatic biochemical markers, despite their diagnostic limitations, may still serve as a useful tool in pre-anesthetic risk assessment, particularly in reproductively active females where hepatic compromise may go undetected until challenged by anesthesia or surgery. Non-esterified fatty acids (NEFAs), widely used in mammals to assess lipid mobilization, have not yet been validated in reptiles but hold promise as future biomarkers to distinguish physiological from pathological fat metabolism. If proven reliable, NEFA monitoring, especially in combination with βHBA, could offer valuable insight into energy balance and hepatic stress in females at risk of dystocia [90].

## 5. Hormonal Treatment

Hormonal therapy is commonly employed in POD once eggs have progressed into the oviduct, typically after shell deposition, when the reproductive tract can respond appropriately to contractile hormones. Before pharmacological stimulation is attempted, basic supportive and husbandry measures should be optimized, as many cases are precipitated or exacerbated by management errors; restoring an appropriate thermal gradient within the species’ preferred optimal temperature zone, minimizing stress, and providing a quiet, suitable nesting area with an appropriate substrate are recommended and may be sufficient to allow for normal oviposition in some non-obstructive cases [45,47]. Patients should also be stabilized with fluid therapy and correction of electrolyte and mineral imbalances, particularly hypocalcemia, together with adequate analgesia and treatment of concurrent disease before any hormonal induction is considered [42,91]. Several authors advocate including parenteral calcium gluconate in medical protocols, frequently administered shortly before oxytocin or AVT in chelonians to enhance oviductal smooth-muscle contractility and improve the response to hormonal treatment, and as part of supportive management in lizards with dystocia [44,59]. Once the animal is adequately supported and obstructive causes have been excluded, oxytocin or can be administered to stimulate effective oviductal contractions via smooth-muscle receptors in both the oviduct and cloacal sphincter, facilitating egg passage. Premature administration, before shell calcification, risks serious complications such as oviduct rupture, hemorrhage, and yolk coelomitis [44,61].

### 5.1. Chelonians

Prior research using 253 female red-eared sliders demonstrated that intramuscular (IM) doses of 10 or 20 IU/kg oxytocin, given up to twice 60 min apart, resulted in egg passage in 100% of cases. The success of lower doses (4 or 5 IU/kg) was 88.9% after two injections [92]. Even at a reduced dose of 4 IU/kg, red-eared sliders, common musk turtles (*Sternotherus odoratus*), and painted turtles (*Chrysemys picta*) exhibited equally high oviposition success after two injections, suggesting broad species applicability [92]. In red-eared sliders with non-obstructive egg retention, a direct comparison of intramuscular and intravenous oxytocin administration demonstrated that both routes were effective in inducing oviposition, although intravenous administration resulted in a faster onset of egg-laying, while intramuscular administration provided a more gradual and clinically manageable response, supporting route selection based on case context and handling constraints [93]. A study involving hawksbill sea turtles (*Eretmochelys imbricata*) found that 0.6–0.8 IU/kg IM oxytocin triggered egg-laying in 8 out of 9 individuals, with oviposition beginning between 17–43 min after administration and completed within 24 h [94]. In contrast, mediterranean tortoises appear to respond to substantially lower doses: in free-ranging Hermann’s tortoises, IM oxytocin at approximately 1.5–3 IU/kg reliably induced oviposition, with first eggs laid 13–68 min after injection and most clutches completed within about 20 min of the first egg [95].

### 5.2. Lizards

Hormonal therapy for POD in lizards typically involves a combined protocol of calcium supplementation followed by oxytocin administration. A well-documented case report in a bearded dragon describes successful oviposition of 14 eggs within 20 h: the protocol included two IM injections of calcium borogluconate (35 mg/kg followed by 50 mg/kg, 30 min apart) followed by a single dose of oxytocin (5 IU, IM) shortly thereafter, with complete resolution and no further retained eggs on radiographs 24 h later. The same individual successfully laid all 38 subsequent eggs uneventfully in the next cycle, suggesting both efficacy and safety of this approach. In contrast, a case series involving leopard geckos (*Eublepharis macularius*) and crested geckos (*Correlophus ciliatus*) demonstrated that medical management with calcium and oxytocin alone was insufficient. In that report, all affected geckos required surgical salpingotomy, followed by supportive therapy, highlighting that while lizards may be candidates for hormonal therapy, success is variable and some species exhibit poor responsiveness to medical protocols [21]. These estradiol-stimulating protocols align with broader clinical recommendations that assert oxytocin should be used within 48 h of shelling, maintained at species-appropriate temperatures, and ideally following calcium correction. Timing is critical to avoid oviductal rupture or ineffective dosing. Though lizard success rates vary, usually cited between 30% and 70%, these peer-reviewed cases confirm that calcium-primed oxytocin therapy can effectively resolve dystocia in small lizards when administered promptly and with supportive care [21,56]. Nonetheless, larger species and delayed cases still frequently require surgical resolution.

### 5.3. Snakes

In snakes, hormonal therapy for dystocia has limited success, with reported effectiveness under 50%, even when administered at the appropriate post-ovulatory stage. The particularly elongated and tortuous oviducts combined with a lower density of oxytocin and AVT receptors likely lowers the effectiveness of oxytocin-based therapies in snakes compared to chelonians or lizards [8]. Intervention must occur within 48–72 h after the onset of oviposition attempts. Delayed administration often results in obstructive dystocia, which carries a high risk of oviduct rupture if oxytocin is used. The typical oxytocin dosing protocol involves administering 5 IU/kg IM or intracoelomically (ICe), with a second dose of up to 20 IU/kg given 6–12 h later if no response is observed [96]. If there is no improvement after two doses, medical therapy should be considered unsuccessful. AVT may be more effective due to its higher receptor affinity, with suggested dosages ranging from 0.01–1.0 µg/kg given IM, subcutaneous (SC), or ICe. However, AVT is rarely available outside of research settings, which limits its clinical application [96,97]. Adjunctive methods can include sedated egg manipulation using propofol (5–10 mg/kg IV) to facilitate cloacal relaxation, followed by manual or endoscopic removal of accessible eggs. In cases where retained eggs are visible but unresponsive to oxytocin, percutaneous ovocentesis may be considered only as a last-resort option, as it carries significant risks of yolk leakage, coelomitis, and oviductal injury and is generally not recommended when surgical management is feasible. This involves aspirating the contents of the egg using a 20-gauge needle under anesthesia to reduce volume and facilitate expulsion. This technique should only be used early in the dystocia process and always with readiness for surgical conversion, given the risk of yolk leakage and subsequent yolk coelomitis [96]. If the snake’s clinical condition deteriorates, evidenced by anorexia, increasing coelomic distension, or signs of sepsis, or if no response is seen after two hormonal treatments, surgical intervention is indicated. Several detailed case reports document successful non-surgical resolution of POD in snakes using hormonal and supportive interventions. A notable study described a 15 kg female albino Burmese python (*Python bivittatus*) with confirmed egg retention; following coelomic fluid therapy, ICe calcium gluconate (50 mg/kg SC, diluted), and 5 IU/kg IM oxytocin, the snake oviposited all 25 eggs within 12 h of a second identical oxytocin dose given after 12 h [98]. This shows effectiveness when medical therapy is initiated within a critical post-ovulatory window and combined with calcium and environmental support. Older veterinary research also notes successful oxytocin use in oviparous snakes, with dosages from 0.4 to 10 IU/kg, achieving egg passage under appropriate conditions [8]. These cases reinforce the principle that oxytocin can be effective in early, non-obstructive dystocia in snakes.

### 5.4. Various

Beyond oxytocin and AVT, treatment with prostaglandins, calcium supplementation, and combination therapies have shown clinical value in treating dystocia. In a case report involving leopard geckos and crested geckos, Di Giuseppe et al. administered IM calcium gluconate at 35 mg/kg, followed by ICe oxytocin, and noted that in some cases additional PGF_2_α was experimented with, facilitating uterine contraction and completion of egg passage [21]. The authors emphasized that prostaglandins must be used cautiously due to limited reptile data, yet when combined with calcium and oxytocin, they helped avoid surgery in small lizards.

Although the literature is scarce for snakes, hybrid approaches have been documented. A Burmese python case incorporated calcium before 5 IU/kg oxytocin IM, followed by a second dose, to facilitate passage of 25 eggs, illustrating that combined protocol principles apply beyond lizards [98,99]. Experimental studies in oviparous lizards have demonstrated that ICe administration of prostaglandin F_2_α at doses between 50–200 µg/kg effectively induces uterine contractions and oviposition when oxytocin alone fails [100].

These cases demonstrate that combined protocols, calcium priming, oxytocin, and prostaglandin administration, can significantly improve outcomes and reduce surgical necessity when applied early in POD. However, if two to three cycles over 48–72 h with combination therapy yield no response or if systemic deterioration ensues, surgical intervention remains the indicated next step [21,96].

## 6. Surgical Intervention Thresholds

### 6.1. General Considerations

Indications for surgery include non-response to oxytocin or AVT therapy after 24–48 h of appropriate dosing, especially in the absence of uterine contractions or egg movement on imaging [47].

Worsening clinical condition, manifested as persistent anorexia, weight loss, lethargy, regurgitation, or weakness despite supportive therapy, should prompt surgical reassessment. In these cases, waiting may increase the risk of egg calcification, oviductal rupture, or yolk peritonitis [1,101].

Imaging findings play a pivotal role. Failure of eggs to progress or reposition on serial radiographs, CT, or ultrasonographic examinations despite appropriate husbandry and medical therapy, prolonged egg retention over several weeks accompanied by clinical deterioration, or excessive oviductal distension with mechanically obstructive eggs (e.g., markedly oversized, fused, or severely deformed eggs wedged within the pelvic canal) are widely regarded as indications to consider surgical management, rather than egg shape alone [44,47,61,64]. However, in chelonians, mildly irregular or malshaped eggs may occur in otherwise uncomplicated clutches and do not, by themselves, constitute dystocia; surgical decision-making should therefore be based on the overall clinical picture and imaging evidence of obstruction or secondary coelomic disease, not morphology in isolation [44,61,64]. Egg retention into late summer or early autumn may reflect physiological preparation for hibernation rather than dystocia. In such cases, otherwise static eggs may still respond to oxytocin or AVT therapy, and surgical intervention is not always indicated [64]. Ultrasound or radiographic signs of dystrophic or malformed eggs, coupled with coelomic effusion or gas shadows, indicate possible oviductal rupture or infection and warrant urgent intervention [47].

Laboratory abnormalities, such as marked heterophilia, hyperuricemia, elevated AST or CK, or hypoalbuminemia, may support the presence of secondary infection, hepatic compromise, or metabolic exhaustion, all of which lower the threshold for surgery [28]. Leukocytosis, especially when accompanied by a left shift or toxic heterophils, is a well-established indicator of systemic inflammation or sepsis in reptiles [102].

Finally, failure to lay despite multiple ovulatory cycles or visible retained follicles across seasons may represent chronic follicular stasis, which often requires ovariosalpingectomy due to fibrosis, adhesions, and risk of neoplasia. Prompt surgical resolution, ideally before systemic decompensation, has been shown to improve survival, recovery, and future reproductive success [44].

Surgical management of dystocia in reptiles varies significantly depending on species anatomy and the reproductive structures involved. In snakes, a ventrolateral coeliotomy, with the skin incision made between the first and second rows of lateral scales, is the standard approach to access the oviducts, avoiding disruption of the ventral scales and allowing for improved post-operative ambulation [62]. A salpingotomy, involving singular or several direct incision(s) into the oviduct to remove retained ova, may be performed if fertility preservation is desired and the contralateral tract appears unaffected. More severe or recurrent cases may warrant a salpingectomy or complete ovariosalpingectomy [103,104].

In lizards with POD, the recommended surgical approach is a ventral paramedian coeliotomy followed by salpingotomy, allowing for direct removal of retained eggs while preserving ovarian tissue when fertility is to be maintained [62]. In cases of POFS, a bilateral or unilateral ovariectomy or ovariosalpingectomy is considered the treatment of choice, effectively resolving the underlying hormonal imbalance and preventing recurrence [105]. In breeding individuals, where fertility preservation is desirable and disease is unilateral, a hemi-ovariosalpingectomy may be performed to remove only the affected tract while maintaining reproductive potential [18]. In cases of advanced reproductive disease with concurrent systemic compromise (e.g., hepatic lipidosis or coelomitis), open coeliotomy is preferred to allow for full exploration, removal of diseased tissues, and thorough lavage [62].

### 6.2. Considerations for Hepatic and Metabolic Derangements

Reptiles with hepatic lipidosis or other forms of hepatic dysfunction may present increased anesthetic risk due to reduced physiological reserve and impaired drug handling. As the liver is the primary site of biotransformation for many injectable anesthetic agents used in reptiles, including alfaxalone and ketamine-based protocols, compromised hepatic function is considered likely to contribute to prolonged recovery times and increased sensitivity to standard anesthetic doses. Prolonged anesthetic effects and delayed recoveries following alfaxalone administration have been reported in reptiles, highlighting the need for cautious dosing and extended monitoring, particularly in clinically compromised individuals. Experimental and clinical studies further indicate that reduced hepatic perfusion and metabolic capacity in reptiles can influence anesthetic depth and recovery characteristics, supporting conservative anesthetic management in patients with suspected hepatic disease [106]. Additionally, hypoalbuminemia, a common consequence of liver disease, may increase the free fraction of protein-bound anesthetic drugs, increasing effective plasma levels, with subsequent risks of toxicity [107]. This is of particular concern with agents like midazolam and propofol, which are highly protein-bound in circulation [108]. Anemic or hypovolemic patients, which are frequently seen in hepatic lipidosis due to anorexia or chronic systemic inflammation, are also less tolerant of vasodilation and hypotension under inhalant anesthesia. Therefore, caution is warranted when selecting both injectable and inhalant agents in liver-compromised reptiles.

Where possible, inhalant anesthesia, particularly sevoflurane or isoflurane, is preferred over injectable protocols in reptiles with compromised hepatic function, as these volatile agents are primarily eliminated via the lungs with minimal hepatic metabolism [101,109]. While these agents can cause peripheral vasodilation and dose-dependent hypotension, this effect is predictable and manageable, and the anesthetic depth can be rapidly titrated or reversed by adjusting inspired concentrations [109]. However, achieving surgical depth with inhalants alone may result in marked respiratory depression or apnea in many reptiles, limiting titration unless controlled ventilation is available. In such cases, a multimodal anesthetic approach, combining low doses of injectable agents with inhalants, can reduce the dose-dependent side effects of individual drugs while improving anesthetic safety and cardiovascular stability. In contrast, injectable agents often rely on hepatic metabolism and may accumulate in reptiles with impaired clearance, leading to prolonged and potentially deeper hypotension. If opioids are desired, agents such as butorphanol (1–2 mg/kg IM q12 h) that are less reliant on hepatic clearance should be used [97]. However, even with inhalants, hepatic perfusion may still be reduced under anesthesia, especially in dehydrated or cold-stressed patients [110].

Adequate hydration is critical to improve hepatic perfusion and drug clearance in lipidosis-affected reptiles. Isotonic fluids like Lactated Ringer’s at 20–30 mL/kg/day SC or IV/intraosseous (IO) are recommended after assessing dehydration level. In cases of hypoalbuminemia or coelomic effusion, colloid administration (e.g., hetastarch 1–2 mL/kg IV) or plasma (10 mL/kg) may be used to support oncotic pressure, though data in reptiles is limited [26]. Reptiles with hepatic lipidosis frequently present anorectic and in a negative energy balance, exacerbating fat accumulation in the liver. Early nutritional support via syringe feeding or assisted tube feeding with high-protein, low-fat reptile diets (e.g., Carnivore, Omnivore or Herbivore Critical Care) helps reverse catabolism [28]. In parallel, supplementation with L-carnitine, widely use in small animal hepatic lipidosis, may offer a promising adjunctive treatment in reptiles, though clinical trials are still needed. A dosage of 250 mg/kg orally daily in reptiles may enhance mitochondrial β-oxidation and support hepatic recovery, but further investigation is warranted [111]. Chronic illness in hepatic lipidosis can result in anemia, hypoalbuminemia, and coagulopathies. Hematologic profiles should be obtained; values like packed cell volume < 15% or albumin < 1.5 g/dL indicate the need for stabilization before anesthesia [26].

**Figure 6:**
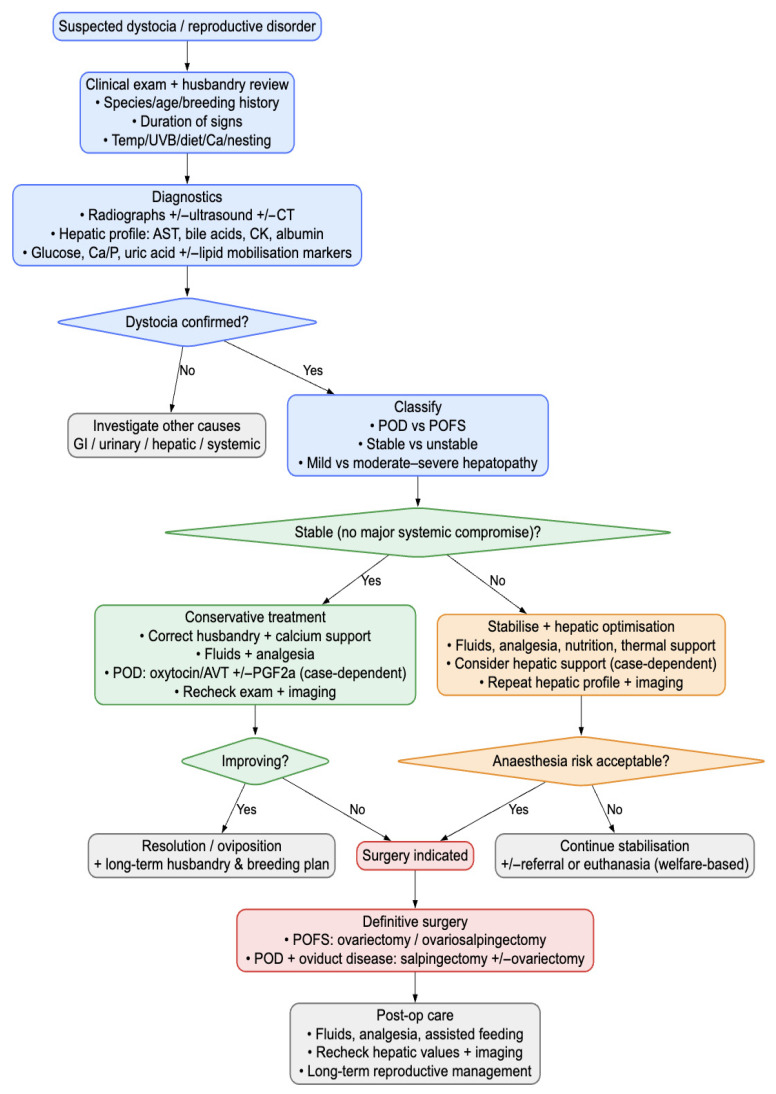
Clinical decision support flowchart for reptile dystocia.

To aid clinicians in applying the surgical thresholds described above, we include a flowchart that integrates patient status, diagnostic findings, and response to medical therapy to guide when surgical management becomes appropriate (Figure 6).

## 7. Future Directions and Clinical Gaps

Despite growing clinical experience and an increase in case documentation, the management of dystocia in reptiles is still limited by several fundamental evidence gaps. One of the most pressing needs is the development of species-specific hormonal therapy protocols. The effectiveness of oxytocin, PGF_2_α, and AVT varies significantly between taxonomic groups, in part due to differences in oviductal receptor expression and endocrine sensitivity [112]. However, most therapeutic protocols remain extrapolated from mammalian practice or isolated case reports, underlining the urgent need for controlled trials in commonly affected reptile species.

Another critical gap lies in the lack of validated liver biomarkers to assess the anesthetic and surgical risk in dystocic reptiles. While hepatic lipidosis and hepatic compromise are common comorbidities, especially in cases of prolonged follicular retention, traditional liver enzymes such as AST, alanine aminotransferase, and bile acids demonstrate high interspecific variability and poor correlation with functional reserve. This makes preoperative risk assessment challenging and inconsistent. Further research is needed to establish species-specific reference intervals and explore the use of additional biomarkers such as NEFA and sorbitol dehydrogenase, which may offer improved diagnostic sensitivity.

Preventative medicine in reproductive care is also underdeveloped. There is currently limited research on pre-emptive support strategies for high-risk breeding females. Evidence from herpetoculture and field ecology suggests that thermal gradients, nesting site quality, and pre-breeding nutritional conditioning all impact reproductive outcomes [13,14]. However, structured studies evaluating prophylactic calcium, vitamin D_3_, or micronutrient supplementation protocols, particularly in captivity, are still lacking.

Another area of inconsistency is the criteria used to determine the need for surgical intervention. Without a standardized scoring system, the decision to move from medical to surgical management is highly variable between clinicians. Similarly, the interpretation of imaging data, such as ultrasound evidence of uterine wall thinning, egg deformation, or pelvic obstruction, remains subjective. The development of validated diagnostic thresholds could guide clinical decision-making and reduce delays that contribute to morbidity and mortality.

In addition to biochemical and hepatic contributors to reproductive failure in reptiles, emerging research suggests that factors such as endocrine-disrupting chemicals (EDCs), reproductive tract microbiota, and trace element imbalances (e.g., selenium, zinc) may also play a role. While not yet well studied in reptiles, EDCs have been shown to impair reproductive endocrinology in alligators and amphibians [113,114]. Moreover, alterations in the reproductive microbiome have been linked to implantation failure in mammals [115]. Similarly, trace minerals influence hepatic antioxidant capacity and reproductive hormone regulation in other vertebrates [116]. These findings support future investigations into their relevance in reptilian dystocia.

## 8. Conclusions

This review reinforces that dystocia in reptiles is not merely a mechanical obstruction but often the clinical manifestation of a complex and self-perpetuating interplay between endocrine dysregulation, hepatic dysfunction, and inappropriate environmental conditions. Hepatic lipidosis, hypoalbuminemia, and altered estrogen metabolism emerge as key mediators of reproductive arrest, particularly in species subject to prolonged vitellogenesis without oviposition. Although calcium-primed oxytocin remains the most widely applied medical protocol, therapeutic success varies considerably across taxa, reflecting underlying differences in receptor pharmacodynamics, metabolic resilience, and reproductive anatomy. Diagnostic advancements, including serial liver function profiling, βHBA and succinate quantification, and high-resolution imaging, present promising avenues for early case stratification and perioperative planning. However, several key gaps remain, including how chronic hepatic lipidosis affects reproductive hormone clearance and receptor responsiveness; whether βHBA and succinic acid can serve as reliable, non-invasive predictors of dystocia severity and anesthetic risk; and how oxytocin pharmacokinetics and receptor expression differ across reptilian lineages. Additionally, the potential for endocrine or nutritional modulation during vitellogenesis to reduce follicular arrest has yet to be fully explored. Addressing these questions will not only refine clinical protocols, but also advance our broader understanding of reproductive energetics and endocrine-immune crosstalk in ectothermic vertebrates.

## Figures and Tables

**Table 1:** Species-specific most common presentations of dystocia in chelonians, associated risk factors, and relevant literature (UVB = ultraviolet B, POFS = pre-ovulatory follicular stasis, POD = post-ovulatory dystocia).

Chelonian Species	Common Name	Type of Dystocia	Key Risk Factors	References
*Testudo graeca*	Greek tortoise	POD due to pelvic obstruction	Narrow pelvis, lack of nesting site, dehydration	[10,44]
*Testudo hermanni*	Hermann’s tortoise	POFS and coelomitis	Persistent follicular development, inflammation, hypocalcemia	[44,50]
*Trachemys scripta elegans*	Red-eared slider	Cloacal prolapse and egg impaction	Water quality issues, inadequate UVB radiation, cloacal muscle weakness	[44,45,49]
*Chelonia mydas*	Green sea turtle	Exhaustion-related egg retention during nesting	Long oviposition, dehydration, human disturbance	[44,51]

**Table 2:** Most common species-specific presentations of dystocia in snakes, associated risk factors, and relevant literature (POD = post-ovulatory dystocia).

Snake Species	Common Name	Type of Dystocia/Reproductive Issue	Key Risk Factors	References
*Python regius*	Ball python	Follicular stasis, retained unfertilized ova, coelomitis	Prolonged follicular phase, improper pairing, low temperature	[10,52]
*Python molurus bivittatus*	Burmese python	Obstructive dystocia, egg retention	Obesity, large clutch size, low calcium, oviposition failure	[53]
*Python sebae*	African rock python	Egg-binding with cloacal prolapse	Egg retention, lack of appropriate nesting, dehydration	[54]
*Lampropeltis getula*	Common kingsnake	POD	Inadequate humidity, inappropriate nesting environment	[45]

**Table 3:** Most common species-specific presentations of dystocia in lizards, associated risk factors, and relevant literature (UVB = ultraviolet B, POFS = pre-ovulatory follicular stasis, POD = post-ovulatory dystocia).

Lizard Species	Common Name	Type of Dystocia/Reproductive Issue	Key Risk Factors	References
*Eublepharis macularius*	Leopard gecko	POD	Lack of UVB radiation, calcium deficiency,	[21,55]
*Correlophus ciliatus*	Crested gecko	POD	Poor husbandry, lack of nesting substrate	[21]
*Pogona vitticeps*	Bearded dragon	POD with large clutch	Hypocalcemia, dehydration, improper thermal and UVB gradients	[56]
*Chamaeleo calyptratus*	Veiled chameleon	POFS	Calcium deficiency, chronic reproductive cycling, photoperiod issues	[57,58]
*Varanus albigularis*	White-throated monitor	Yolk coelomitis from retained follicles	Failure to oviposit, prolonged retention	[43]

## Data Availability

The original contributions presented in this study are included in the article. Further inquiries can be directed to the corresponding author.

## Abbreviations

AST	Aspartate aminotransferase
AVT	Arginine vasotocin
βHBA	Beta-hydroxybutyric acid
CK	Creatine phosphokinase
CT	Computed tomography
EDC	Endocrine-disrupting chemicals
ERα	Estrogen receptor alpha
ERβ	Estrogen receptor beta
ICe	Intracoelomic
IU	International units
IO	Intraosseous
IM	Intramuscular
IV	Intravenous
NEFA	Non-esterified fatty acids
PGF_2_α	Prostaglandin F2 alpha
POFS	Pre-ovulatory follicular stasis
POD	Post-ovulatory dystocia
SC	Subcutaneous
UVB	Ultraviolet B radiation
